# Wilson’s disease: Food therapy out of trace elements

**DOI:** 10.3389/fcell.2022.1091580

**Published:** 2022-12-21

**Authors:** Wen-Jie Li, Huan-Ling Chen, Bin Wang, Lei Yao, Xiao-Ping Wang

**Affiliations:** ^1^ Department of Neurology, Jiading Branch of Shanghai General Hospital, Shanghai Jiao Tong University School of Medicine, Shanghai, China; ^2^ Ningbo First Hospital, Ningbo, China; ^3^ International Academician Station JiangQiao, Shanghai, China

**Keywords:** Wilson’s disease, trace elements, metal, copper, protein, food therapy

## Abstract

Hepatolenticular degeneration, also known as Wilson’s disease (WD), is an autosomal recessive inheritance nervous disorder of copper metabolism. The treatment of hepatolenticular degeneration emphasizes the combination of medical therapy and dietary therapy, such as a high zinc, low copper and sulfhydryl rich diet. Food therapy of WD based on trace elements is presented in this paper.

## 1 Introduction

**FIGURE 1:**
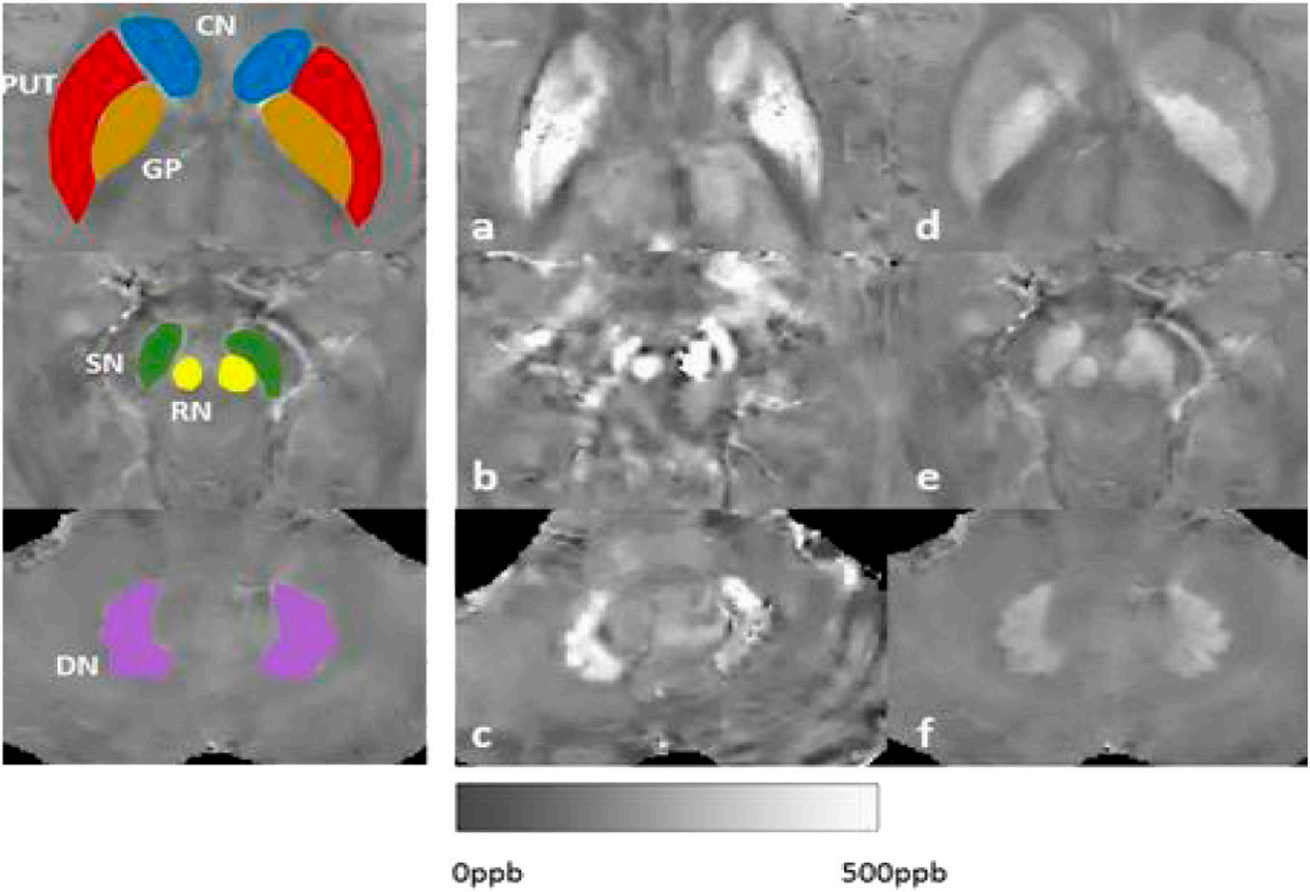
Our results of MRI-QSM signals: iron/copper related Magnetic Susceptibility in the Deep Gray Matter Nuclei of Wilson’s Disease. Note: CN: caudate nucleus; GP: globus pallidus; PUT: putamen; SN: substantia; RN:rednucleus; DN: dental nucleus.

Hepatolenticular degeneration, also known as Wilson’s disease (WD), is an autosomal recessive inheritance disorder of copper metabolism with a global prevalence of about 1/100,000–3/100,000 (Czlonkowska et al., 2018; Cai et al., 2022). Its pathogenic gene is the ATP7B gene, which encodes the copper-transporting P-type adenosine triphosphatase that catalyzes the binding of copper ions to ceruloplasmin so that copper ions can be secreted into bile from hepatocytes and excreted *via* biliary tract and intestinal tract (Nishito and Kambe, 2018). In case of the absence or decreased function of this enzyme, the excretion of copper ions *via* bile is reduced, resulting in the deposition of copper ions in the liver. Furthermore, copper ions can be secreted into the blood and deposited in the brain, kidney, cornea and other organs and tissues, leading to liver damage, central nervous system abnormalities and mental symptoms. Considering that hepatolenticular degeneration is a typical disorder of copper metabolism, a copper-restricted diet has long been considered one of the important measures for the treatment of hepatolenticular degeneration. Besides to investigate the results of copper/iron contents in blood, urine, tissues, and cerebrospinal fluid (CSF), we have investigated MRI-Quantitative susceptibility maps (QSM) signals: iron/copper related Magnetic Susceptibility from the brain to other organ, such as liver situations (Figure 1).

The American Association for the Study of Liver Diseases (AASLD) guidelines suggest that patients with hepatolenticular degeneration should avoid eating food and water containing high concentrations of copper, especially in the first year of copper excretion therapy (Saroli Palumbo and Schilsky, 2019). The European Association for the Study of the Liver (EASL) clinical practice guidelines, which refer to those proposed by the AASLD, also put forward some suggestions on the diet of patients with hepatolenticular degeneration on the restriction of copper intake (Saroli Palumbo and Schilsky, 2019). However, given the rarity of the disease and the diversity of its clinical manifestations, there are no published randomized controlled trials on the disease, resulting in uncertainty about the benefit of a copper-restricted diet for patients with hepatolenticular degeneration. In addition, a long-term copper-restricted diet is considered to affect patients’ quality of life and induce or aggravate malnutrition. For these reasons, WD treatment based on food therapy is presented in this paper for reference.

## 2 Drinking water

Patients with WD are advised to prohibit drinking water containing high concentrations of copper. For a healthy adult, a daily intake of copper content of 0.5–2 mg is required. Experts believe that the copper content in drinking water can reach 0.2 mg/L. If a person drinks 1000 ml of water a day, 0.2 mg of copper will be ingested through drinking water. Therefore, pure or distilled water is recommended, particularly during initial therapy (Brewer and Yuzbasiyan-Gurkan, 1992). It was also proposed that after the first year of copper excretion therapy, if the patient is taking an appropriate anticopper agent in standard doses, the general diet can not be strictly controlled, including drinking water (Brewer and Yuzbasiyan-Gurkan, 1992). In fact, food sources supply less content of copper to the organism, and sometimes copper comes more from inorganic sources such as copper plumbing or copper kitchenware, which should be avoided.

## 3 Foods

Common foods listed by Wilson’s Disease Association, British Liver Trust, American Liver Foundation, the Mayo Clinic and China Hepatolenticular Degeneration Center (http://www.wilson-disease.org/) include chocolate, dried fruits, animal livers, mushrooms, nuts, multigrain bread and shellfish. As far as the daily diet management of patients with hepatolenticular degeneration is concerned, it is difficult to justify the recommendation of avoiding most of the more copper-rich foods, as they need a large amount of intake to make the absorbed copper higher than the normal level. The European Dietary Guidelines recommend a general adult dietary intake of 1.3 mg d^−1^ for females and 1.6 mg d^−1^ for males (Jing et al., 2022). If the daily intake of copper is 1.7 mg by consuming copper-rich foods, then a normal person needs to consume 500 g whole wheat bread (with an average copper content of 3.4 mg kg^−1^), 1932g pure milk (with a copper content of 0.88 mg kg^−1^), 1954g broccoli (with a copper content of 0.87 mg kg^−1^) or 2125 g salmon meat (with a copper content of 0.8 mg kg^−1^) every day (Wang et al., 2020). This does not even take into account the absorption rate of copper. Assuming that the absorption rate of copper in the diet is about 36%, then the required intake of the above foods is more than that (Taylor et al., 2020). However, oysters contain 16 mg kg^−1^ copper and sheep liver 157 mg kg^−1^copper (Wang et al., 2020). Even with the lowest absorption rate of copper ions, a standard consumption of shellfish and animal liver (120 g) can make the intake of copper ions exceed the standard or even reach a very high level (Taylor et al., 2020). It is, therefore, recommended to abstain from shells, nuts, animal offals and fat, seafood and other foods with high copper content, instead, it is advisable to eat polished rice, white flour, light vegetables, fruits, lean meat, poultry meat, eggs and scaly freshwater fish with low copper content. Nevertheless, there are a few points worth noting:①Scallop in marine shellfish is recommended by Brewer because its copper content is about 1/2 that of eggs (Brewer and Yuzbasiyan-Gurkan, 1992); ②Most beans, such as red beans and black beans, contain very high levels of copper, but the copper content of soybean and tofu measured by Brewer is not high (Brewer and Yuzbasiyan-Gurkan, 1992). Eight of the 37 soybean samples tested in China have high copper content (Yao and Wang, 2002)^,^ and there may be regional differences besides the sampling error, which suggests that the local copper content should be the standard for patients living in a certain area. ③ A lot of materials reflect that some animal fats are not high in copper, but WD is prone to cholelithiasis and cholecystitis, Zischka’s group demonstrated that high-calorie diet damages the liver in WD rats (Einer et al., 2019), so a high-fat diet, especially saturated fatty acids, should be avoided.

Brewer et al. also proposed that patients beginning decoppering therapy should avoid liver and shell fish, but otherwise may be on a completely unrestricted diet. After decoppering, patients should eat shell fish no more often than once a week, and no more than half an ounce of liver once a month (Brewer and Yuzbasiyan-Gurkan, 1992).

## 4 Traditional Chinese herbs’ diet therapy

Traditional Chinese Medicines with functions of cholagogue and promoting dieresis can be used to ameliorate the excretion of copper by means of bile, feces and urine. WD Decoction created by Yang RM had been proved to be effective for WD (Yang, 2007). Currently by the authors’ clinical experience, the total treatment scheme integrated Chinese and Western medicine is recommended for WD, especially for early stage patients and in the maintenance therapy stage. One of the prescription principles of WD decoction created by Yang RM et al. is that traditional Chinese medicines are high in zinc and low in copper (Yang, 2015), such as cinnamon, tuckahoe, polyporus, rhubarb, cortex phellodendri, lysimachiachristinae hance, andrographis paniculata, turmeric, radix isatidis, fructus gardeniae and alismatis rhizoma, which belong to traditional Chinese medicines for treating liver diseases. There are many questions about American ginseng. It is generally believed that the content of copper in American ginseng is low, but the content of copper and zinc in ginseng produced in China is difficult to reach the same level after several groups of measurements (Cao et al., 1993). Medicinal and edible products, such as dried tangerine peel, licorice, wolfberry, chrysanthemum, seabuckthorn, cinnamon, dark plum, almond, nutmeg, amomum villosum and siraitiagrosvenorii are basically low in copper and high in zinc. Of course, the origin and other factors can not be ignored. However, western scholars have more doubts about treating WD with traditional Chinese medicine.

## 5 Copper-bound natural substance

In 1807, researchers detected copper in animals. In 1878, ceruloplasmin was first isolated from the octopus. Some crustaceans, such as snails and shrimps, had blue “blood” because copper (ceruloplasmin) replaced iron (hemoglobin). In 1871, researchers detected zinc from animals. Zinc has the effect of resisting copper and promoting copper excretion. Currently, penicillamine, the main drug for excreting copper in clinical practice, was first found in human urine, which is a natural biological metabolite or derivative. There are other natural complexing agents such as rhodotorulic acid, the metabolite of *Streptomyces* deferoxamine, and marine shrubs also has weak copper excretion ability. It was thought that WD was a disorder of amino acid metabolism in protein because there were a large number of amino acids and proteins in urine besides high copper, most of which were proline and citrulline. At the same time, it was observed clinically that babies who were mainly fed milk for a long time were prone to low copper anemia, so it was inferred that WD should have more high protein such as drinking milk. Theoretically, it is possible to chelate copper if it is rich in phenolic hydroxyl (tyrosine, phenylalanine) or sulfhydryl (cysteine).

In the process of using lifelong copper excretion drugs in patients suffering from WD, some other metals are also discharged. Besides low zinc, low calcium, low iron, low phosphorus, etc. often occur. Therefore, dietary intake of the following metals should be considered: ① For iron deficiency, more iron-rich foods should be taken in normal times, such as animal muscle, egg yolk, lean meat and beans; celery and bean sprouts in vegetables; hawthorn, apricot, peach and grape in melons and fruits. In addition, attention should be paid to providing an acidic environment for the stomach, such as the appropriate use of vitamin C and vinegar, to facilitate the digestion and absorption of iron. ② For zinc deficiency, zinc supplementation from medication is also an inexpensive way. Foods rich in zinc include lean meat, eggs, fresh fish and milk. The bioavailability of zinc from animal food is higher than that from plants. ③ For calcium deficiency, milk contains the most calcium, and others such as eggs, beans and bean products are the next best. ④ For phosphorus deficiency, more soybeans, cereals, plums, grapes, chicken, potatoes, eggs, etc. can be consumed. ⑤ For manganese deficiency. Tea is rich in manganese in food, rice, millet, flour and sweet potato are rich in manganese in grain, while apples, oranges, apricots and pears are rich in manganese in fruit. When choosing related foods with the above trace elements, we also need to take their copper content into consider.

It should be noted that up to now, there are neither a large number of randomized controlled clinical studies on the copper-restricted diet in patients with hepatolenticular degeneration nor sufficient evidence that a copper-restricted diet can benefit patients with hepatolenticular degeneration. Given that the absorption of copper decreases with increasing dietary copper content, it has been suggested that the “copper-rich diet” in no way significantly increases the absorption of copper (Xu and Fan, 2022). However, there is consensus among researchers that crustaceans, livers, nuts, chocolate and mushrooms should be banned, and that the copper content of foods should be determined in the context of local conditions. Despite the beneficial effects of copper-restricted diets for patients with hepatolenticular degeneration, the distress caused by such diets cannot be ignored, which may easily lead to malnutrition or nutritional imbalance. For patients, nutrition is always an important issue, especially high protein for all WD patients, except for the prodrome and true hepatic coma. Nutritional intervention for patients with hepatolenticular degeneration, especially liver type, should focus on how to improve nutritional status, prevent malnutrition and reduce fluid retention due to hypoproteinemia. A daily diet rich in carbohydrates, protein, fat and multivitamins and balanced in nutrition is of great importance for the health of patients with hepatolenticular degeneration (Li et al., 2016; Choi et al., 2022; Lee et al., 2022).
